# Analytical Modeling of Debonding Mechanism for Long and Short Bond Lengths in Direct Shear Tests Accounting for Residual Strength

**DOI:** 10.3390/ma14216690

**Published:** 2021-11-06

**Authors:** Amir Mohammad Mirzaei, Mauro Corrado, Alberto Sapora, Pietro Cornetti

**Affiliations:** Department of Structural, Geotechnical and Building Engineering, Politecnico di Torino, Corso Duca degli Abruzzi 24, 10129 Torino, Italy; mauro.corrado@polito.it (M.C.); alberto.sapora@polito.it (A.S.); pietro.cornetti@polito.it (P.C.)

**Keywords:** debonding, composite joints, friction, fiber-reinforced cementitious matrix (FRCM) composites, pull-push test, shear lag model, pullout test

## Abstract

Interfacial debonding in fiber-reinforced composites is a common problem, especially in external strengthening techniques. This investigation aims to determine the load during debonding, and discusses two practical design parameters for direct shear tests, which are commonly used to assess the mechanics of debonding. In this study, three different bond-slip cohesive laws and one finite fracture mechanics approach are considered to investigate debonding in direct shear tests by taking the effect of residual strength into account. For each model, load during debonding and its maximum value are given by closed-form expressions, which are then checked against experimental data reported in the literature. It is shown that using the interfacial mechanical properties extracted from one geometry, the debonding load of tests with different bond lengths and widths can be predicted without any fitting procedure. Moreover, effective bond length formulae are suggested for each model; one is the straightforward extension (accounting for residual strength) of a formula available in the Standards. The results illustrate the importance of considering residual strength in direct shear tests, even at debonding onset, with its effect being nonetheless higher for long bond lengths.

## 1. Introduction

One possible solution to increase or restore the load bearing capacity of an existing structure is to apply external reinforcements. Fiber Reinforced Polymers (FRPs) possess high strength to weight ratios. Thus, FRP plates and sheets have been widely used for structural retrofitting in the last decades, also because of their easy and quick application [1]. However, FRPs have some drawbacks, e.g., high thermal mismatch between the structure and epoxy resins as well as poor fire resistance. To overcome such problems, recently, fiber-reinforced cementitious matrix (FRCM) composites have been considered, and their application seems promising [2,3].

One of the major issues in this strengthening technique is to prevent the debonding of the reinforcement from the structure. Thus, investigation about the debonding of FRP/FRCM from the structure seems crucial, as it may define the load-bearing capacity of the structure. The most common experimental test to analyze debonding is the direct shear test, sometimes named the pull-push test, in which the load directly pulls up the FRP or FRCM, and the block is pushed using the reaction force of a fixed frame. It can be argued that the interface is mainly subjected to shear, and the effect of peeling stress can be neglected, as it has been shown that the contribution of peeling stress is highly localized for this test [4]. On the other hand, a considerable number of investigations can be found in the literature that have successfully applied the one-dimensional shear-lag model [5]. In this paper, the shear-lag model is employed in four different approaches in order to study the debonding of the reinforcement from the substrate. All the approaches take the effect of residual strength into account. This is an important task, especially for FRCM strengthening systems. Direct shear tests on FRCM reinforced structures are characterized by a load vs. displacement curve where the load does not fall to zero but, rather, to a constant value. In FRCMs, debonding occurs typically by pulling off the fiber net from the cementitious matrix, and friction occurs between the two components. As the effect of friction is considered as traction in calculations, the shear stress attained for large slips is called residual strength.

The scientific literature about the subject is vast. In the following, we provide a brief review of studies which are closely related to the analysis we perform in the paper.

Regarding analytical investigations, a bilinear cohesive law (i.e., not considering any residual strength) was employed by Yuan et al. [6] to model the debonding process of FRP-to-concrete joints. The same problem was addressed using linear-exponential softening law by Cornetti and Carpinteri [7], and then via exponential softening by Biscaia et al. [8]. The analytical solution for a trilinear bond-slip law, i.e., considering the residual strength, was first provided by Ren et al. [9], where the authors analyzed the debonding comportment of grouted rockbolts. However, the validity of the analyses carried out in [6,9] are restricted to sufficiently large bond lengths. Extensions to short bond lengths were provided by Cornetti and Carpinteri [7] and Caggiano et al. [10] in the absence of residual strength and, more recently, by Vaculik et al. [11] by considering their effects. D’Antino et al. [12] also applied a trilinear cohesive crack model to experimental data on polyparaphenylene benzobisoxazole (PBO) FRCM composites [13]. On the other hand, two simpler constitutive interface laws were employed in refs. [14,15] to address the same problem. Finally, note that Cornetti et al. [16] used a fracture criterion called finite fracture mechanics (FFM) [17] to determine analytically the delamination load in the pull-push test. They illustrated that FFM results are close to those obtained by the cohesive crack model. Grande et al. [18] developed an analytical model to study the debonding of FRCM strengthening systems considering two interfaces for mortar layers.

Regarding numerical studies, a nonlinear finite element method was used by Pham and Al-Mahaidi [19] to predict maximum debonding load, strain field and bond-slip curve for experimental tests of single-lap pull-push. Biscaia et al. [20] compared the debonding response of FRP-to-concrete joints for various constitutive interface laws by exploiting finite differences. Two different approaches for both 2D and 3D finite element methods were employed by Barbieri et al. [21] to simulate debonding process for FRP-to-concrete tests conducted by the authors. Recently, Carloni et al. [2] used a 3D cohesive crack model for single-lap direct shear tests of FRCM-concrete joints. Also, Muñoz-Reja et al. [22] implemented the FFM in a finite element commercial code and compared their analysis with the experimental results described in [23]. The debonding mechanism in the pull-push test for FRP-to-concrete joints was analyzed by Zhang et al. [24] using a numerical approach called bonded-particle model. Additionally, Ciampa et al. [25] analyzed the bond behavior of the pull-push test by utilizing two different approaches in 3D finite element method. A nonlocal model was developed by Marfia et al. [26] according to the weighted spatial averaging approach to study debonding of FRP from concrete.

Concerning experimental analyses, Bizindavyi and Neale [27] used an experimental approach for the single-lap direct shear test to investigate the bond behavior of FRP-to-concrete joints, and then modeled their problem using a theoretical approach. Ali-Ahmad et al. [28] examined the bond performance of FRP-to-concrete using the digital image correlation approach. Experimental research on the effectiveness of FRCM for the strengthening of concrete was performed by D’Ambrisi [29]. The authors claimed the existence of effective bond length (which is also discussed in this paper) and final slip. A comparison between single- and double-shear tests was conducted by Sneed et al. [30] experimentally. The authors argued that the maximum load is slightly higher in the single-lap test. Apart from investigating the effect of bond length in the pull-push test, Ombres [31] studied the influence of service temperature on behavior of FRCM-to-concrete bonds. In an interesting study, Biscaia et al. [32] investigated the effect of residual bond strength on debonding behavior by applying an external pressure in double-shear tests. The authors demonstrated the high effect of residual bond strength on the interface performance. An analysis of the debonding mechanism of FRCM with different fibers, i.e., glass, carbon, or steel, and two different types of cement, was conducted by D’Antino et al. [33]. Also, D’Antino et al. [34] studied the effect of concrete strengths and surface preparations of concrete on the failure mechanism of the FRCM. Recently, a modified double-pull shear setup was introduced by Mukhtar and Shehadah [35] to determine the bond performance of FRP-to-concrete joints. It is worthwhile to note that, recently, the fatigue performance of FRCM composites has been receiving a lot of attention [36,37].

In the next section, the mathematical modelling of the problem will be described, and closed-form expressions for the load values during the debonding process, for the maximum debonding load vs. bond length, as well as for the effective bond length, will be presented according to three different interface cohesive laws and one finite fracture mechanics approach. Theoretical predictions will be validated and discussed in Section 3. Finally, conclusions are drawn in Section 4.

The goal of the present work is to determine the load during debonding in direct shear tests according to the four above-mentioned models, along with the two most relevant design quantities, i.e., the maximum transferable load and the effective bond length. The emphasis is placed on the residual strength—usually not considered in previous works on the subject—and its effect on the structural response. The main novelties of this research are the analytical solutions for the Dugdale Model (DM) and the rigid finite fracture mechanics model (RF). Although the two other models (the equivalent-linear elastic brittle interface model (EL) and the rigid-linear softening one (RL)) were proposed recently [14,15], they are reported here for the sake of comparison. Moreover, for the EL model, the analytical expression of the maximum debonding load vs. bond length is original, and the effective bond length formula differs from that available in the literature.

Finally, note that in the present paper, we only considered the direct shear test, which is the most common for FRP/FRCM reinforcements. However, other kinds of tests exist which are able to assess the bonding properties of reinforcements (see, e.g., [38]). Although worthy of investigation, they require proper modelling that goes beyond the scope of the present analysis.

## 2. Mathematical Modeling

**Figure 1:**
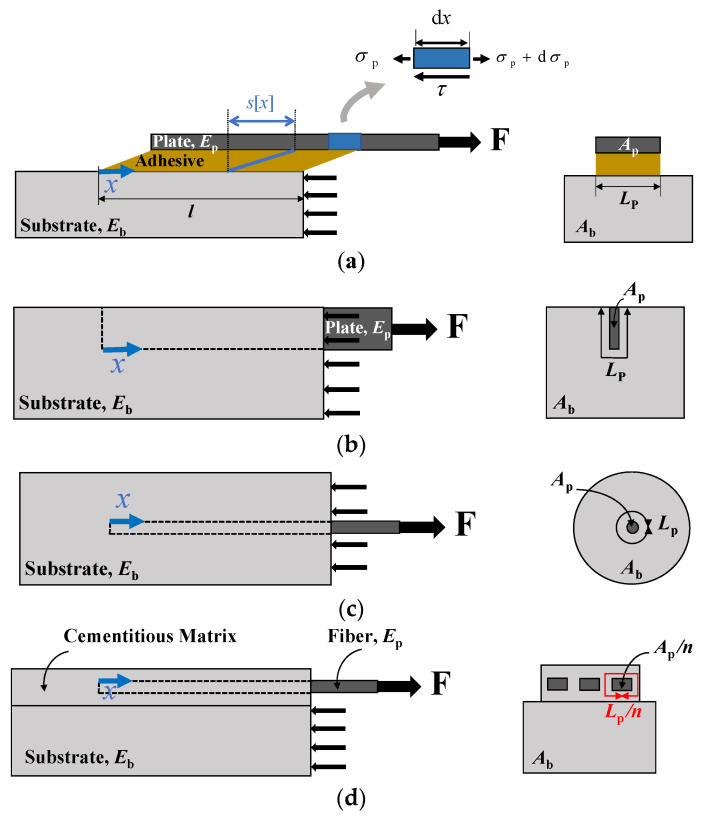
Schematic view of the direct shear tests for: (**a**) externally bonded FRP plate; (**b**) NSM reinforcement; (**c**) embedded bar; (**d**) FRCM strengthening system with *n* = 3 longitudinal fibers. For the sake of simplicity, details for the adhesive layer (enlarged) are given only in (**a**) and proper constraints to avoid block uplifting are not drawn.

The model we are going to develop is a one-dimensional shear-lag model, according to which the interface has only shear stresses and the reinforcement is subjected only to axial loads. Of course, more refined one-dimensional models can be built, modeling, for instance, the reinforcement as a beam and considering peeling stresses along the interface. However, for the geometry at hand, the shear-lag (sometimes called Volkersen’s [5]) model is sufficient for our purposes. Its simplicity allows one to apply it easily to a variety of strengthening systems (see Figure 1): FRP plate, near surface mounted (NSM) reinforcement, embedded bar, and FRCM strengthening system. Provided that the total area of the reinforcement and its perimeter vary from case to case, the equations we are going to derive hold for all the geometries. For the sake of simplicity, in the following figures, we will just illustrate the geometry (a); on the other hand, experimental comparisons will be provided for experiments on FRCM test (d), where the effect of the residual strength—the key feature in the present investigation—is shown to be higher. In this section, the governing equation of the problem is first determined. Then, three different constitutive interface laws are employed, i.e., the equivalent-linear elastic brittle interface model (EL), the Dugdale Model (DM) and the rigid-linear softening model (RL). A fourth fracture mechanics approach, i.e., the rigid finite fracture mechanics model (RF), is also presented. The main novelty is that all models account for the presence of residual strength, and attention is focused on its effect on structural response.

For the first three models, we first solve the corresponding differential equation providing the stresses along the interface. The fourth model is somewhat different, being mostly based on linear elastic fracture mechanics. Thus, it does not require knowledge of interfacial stresses. Then, for all the models, the load during debonding is calculated and the two most relevant important design parameters, i.e., the maximum debonding load and the effective bond length, are computed.

In order to derive the governing equation of the problem, the horizontal equilibrium equations for an arbitrary element of the reinforcement, as well as for the overall reinforcement and substrate system, are needed (see Figure 1a):

(1) $$A_{p}\;d\sigma_{p}-\tau \;L_{p}\;dx=0$$

(2) $$\sigma_{p}\;A_{p}+\sigma_{b}\;A_{b}=0$$

where the subscripts p and b are representative of the reinforcement (the FRP *plate* for the geometry (a)) and of the *block* (substrate), respectively. Parameters *A*_p_ and *A*_b_ represent the cross-sectional area of the reinforcement and the block, while the bonded perimeter is expressed by *L*_p_. We assume that the components follow a linear elastic behavior. Denoting the normal stress as *σ*, Young’s modulus as *E*, and the displacement along the longitudinal axis *x* as *u*, we have *σ_i_* =*E_i_* (d*u_i_*/d*x*), *i* = p, b while the shear stress along the interface between the substrate and the reinforcement is *τ*. Some analytical manipulations lead to the following governing second order differential equation:

(3) $$\frac{d^{2}s}{dx^{2}}-L_{p}\frac{1+\rho }{E_{p}\;A_{p}}\tau [s]=0$$

where *ρ* *= E*_p_ *A*_p_/*E*_b_ *A*_b_ is the mechanical fraction of reinforcement and *s* is the relative longitudinal displacement between reinforcement and block, i.e., *s* = *u*_p_ − *u*_b_. The normal stress in the reinforcement is:

(4) $$\sigma [s]=\sigma_{p}[s]=\frac{E_{p}}{1+\rho }\frac{ds}{dx}$$

For details see, e.g., [16]. In Equation (3), the bond-slip law, *τ*[*s*], is seen as a property of the bonding system, i.e., a constitutive (cohesive) law for the interface. In the following subsections, different models are introduced and employed to analyze the debonding of the reinforcement. Note that they are all based on three parameters, being univocally defined by the fracture energy, G_c_, the shear strength, *τ*_c_, and the residual strength, *τ*_r_.

Regarding the residual strength, it should be noted that it is a mechanical parameter that takes into account, at the macroscale, different mechanisms occurring at the microscale. Among them, stick-slip can be frequently observed in pull-out testing. This phenomenon is typically attributed to the interlocking between the fiber and matrix, resulting in an increase of the load till a critical value. The fiber then slips causing a drop in load-displacement curve (see [39,40,41]). Clearly, the proposed models are not able to catch such mechanisms, but rather, an idealized, averaged, mechanical behavior. Also, it is assumed that the residual strength (friction) has a constant distribution along the debonded zone, assuming the same value for multiple fibers. However, although the use of the residual strength represents an oversimplification of the interfacial behavior, it will be shown that the proposed models are able to catch the basic features of the problem at hand with reasonable accuracy.

### 2.1. Equivalent-Linear Elastic Brittle Interface Model (EL)

**Figure 2:**
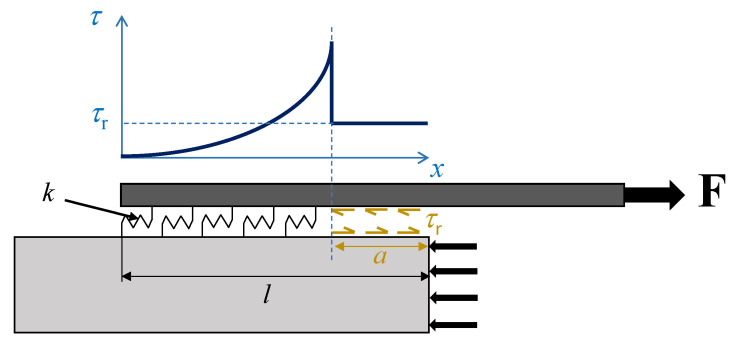
Shear stress field along the interface when the debonding crack has length *a*.

Based on the Equivalent-Linear Elastic Brittle Interface Model, the interface can be considered as a bed of linear springs with a stiffness equal to *k*. The constitutive interface law is presented in Equation (5):

(5) $$\tau [s]=\left\{ \begin{array}{l} k\;s,\;\;\;\;\;s\leq s_{f}\\ \tau_{r},\;\;\;\;s>s_{f} \end{array}\right.$$

where *s*_f_ is the *final* relative displacement, i.e., the displacement corresponding to the drop of the shear stress drops to the residual strength. As the governing equation of the problem is a second-order differential equation (Equation (3)), two boundary conditions are needed to determine the relative displacement, *s*. According to Figure 2 and Equation (4), boundary conditions for the problem can be imposed as follows:

(6) $$\sigma \left[0\right]=0\to s^{\prime }\left[0\right]=0$$

(7) $$\sigma \left[l-a\right]=\frac{F-\tau_{r}\;a\;L_{p}}{A_{p}\;}\;\;\;\to \;\;s^{\prime }\left[l-a\right]=\frac{1+\rho }{E_{p}\;A_{p}\;}\left(F-\tau_{r}\;a\;L_{p}\right)$$

where *a* is the debonded (crack) length with constant stress distribution equal to *τ*_r_. From these boundary conditions, the relative displacement field results:

(8) $$s\left[x\right]=\left(F-a\;L_{p}\;\tau_{r}\right)\sqrt{\frac{1+\rho }{E_{p}\;A_{p}\;L_{p}\;k}}\;\;\mathrm{csch}\left[\left(l-a\right)\sqrt{\frac{k\;L_{p}(1+\rho )}{E_{p}\;A_{p}}}\right]\cosh\left[x\sqrt{\frac{k\;L_{p}(1+\rho )}{E_{p}\;A_{p}}}\right]$$

The corresponding stress is obtained multiplying Equation (8) by the interface stiffness *k*. The maximum is achieved at the edge between the undamaged zone and the frictional region, i.e., at *x* = *l* − *a*:

(9) $$\tau_{\max}=k\;s\left[l-a\right]=\left(F-a\;L_{p}\;\tau_{r}\right)\sqrt{\frac{k(1+\rho )}{E_{p}\;A_{p}\;L_{p}}}\;\coth\left[\left(l-a\right)\sqrt{\frac{k\;L_{p}(1+\rho )}{E_{p}\;A_{p}}}\right]$$

**Figure 3:**
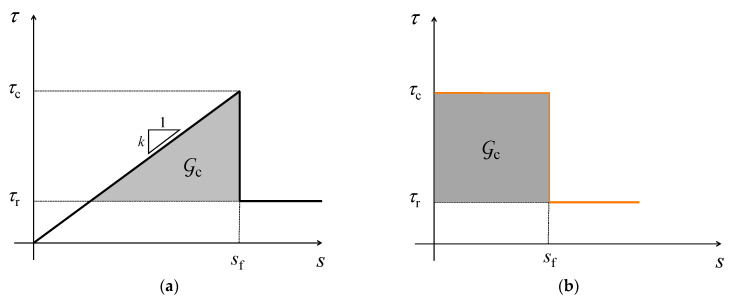

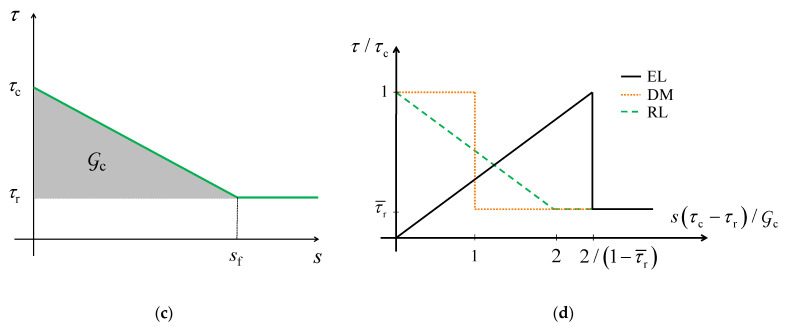
Interface cohesive laws according to: (**a**) Equivalent Linear Elastic Brittle Interface Model (EL); (**b**) Dugdale Model (DM); (**c**) Rigid Linear Softening Model (RL). Their comparison in dimensionless form is in sub-figure (**d**).

Equations (8) and (9) hold for any *k* value and will be exploited later in Section 2.4. On the other hand, we named the present model as *Equivalent*, since in the linear elastic brittle interface model, interface stiffness is an independent parameter characterizing the interface, while in the EL model, *k* depends on the other interface properties, see Equation (11) below. In fact, according to the EL model (see Figure 3a), the stiffness *k* and the “final” displacement *s*_f_ are functions of G_c_, *τ*_c_ and *τ*_r_ according to:

(10) $$s_{f}=\frac{2\;\tau_{c}\;G_{c}}{{\left(\tau_{c}-\tau_{r}\right)}^{2}}$$

(11) $$k=\frac{{\left(\tau_{c}-\tau_{r}\right)}^{2}}{2\;G_{c}}$$

The bond-slip response of the interface, *τ*–*s*, based on the EL model, is depicted in Figure 3a.

To justify the reason for considering the dark area as the fracture energy of the interface, we can simply evaluate the crack closure work for a vanishing step. The stress required to close the crack is *τ*_c_ − *τ*_r_, while the displacement is (*τ*_c_ − *τ*_r_)/*k* corresponding to the dark triangle in Figure 3a, see also [15,42,43]. Analogously, the strain energy release rate G is the elastic energy released by the spring at the crack tip. Hence:

(12) $$G=\frac{{\left(\tau_{\max}-\tau_{r}\right)}^{2}}{2\;k};\;G_{c}=\frac{{\left(\tau_{c}-\tau_{r}\right)}^{2}}{2\;k}$$

Propagation occurs as G reaches G_c_. According to Equation (12), this is tantamount to state that crack grows when *τ*_max_ = *τ*_c_. Before proceeding, it is convenient to normalize the quantities at hand, introducing a reference load and length. The reference load is:

(13) $$F_{c}^{\infty }=\sqrt{\frac{2\;G_{c}\;E_{p}\;A_{p}\;L_{p}}{1+\rho }}$$

It can be proven that, in the absence of a residual strength, $F_{c}^{\infty }$ represents the maximum admissible debonding load, usually achieved for infinite or sufficiently large bond length, independently of the bond-slip law shape, i.e., it depends on the fracture energy only, see e.g., Cornetti et al. [16].

The reference length can be defined as:

(14) $$l_{\mathrm{ch}}=\frac{F_{c}^{\infty }}{\tau_{c}\;L_{p}}=\frac{1}{\tau_{c}}\sqrt{\frac{2\;G_{c}\;E_{p}\;A_{p}}{(1+\rho )L_{p}}}$$

From Equation (14), it is clear that *l*_ch_ can be seen as the bond length that endures $F_{c}^{\infty }$ if the interfacial shear stress distribution is constant and equal to the interface shear strength, *τ*_c_.

We can now introduce the dimensionless auxiliary variables as:

(15) $$\bar{F}=\frac{F}{F_{c}^{\infty }},\;\lambda =\frac{l}{l_{\mathrm{ch}}},\;\alpha =\frac{a}{l_{\mathrm{ch}}},\;{\bar{\tau }}_{r}=\frac{\tau_{r}}{\tau_{c}}$$

By exploiting Equation (9), (12) and (15), the load during the debonding process is achieved as:

(16) $$\;\;\bar{F}=\frac{\tanh\left[\left(\lambda -\alpha \right)\left(1-{\bar{\tau }}_{r}\right)\right]}{1-{\bar{\tau }}_{r}}+\alpha \;\;{\bar{\tau }}_{r}$$

#### 2.1.1. Maximum Load vs. Bond Length

Maximum load during debonding can be considered as one of the most important parameters for design purposes, as it determines the final load-bearing capacity of the joint. By inspection of Equation (16), it can be easily verified that the maximum load,$F_{c}^{}$, is achieved at the crack onset (*a* = 0) for bond lengths lower than a limit value, i.e., for *λ* < *λ*_lim_, where:

(17) $$\lambda_{\lim}=\frac{1}{1-{\bar{\tau }}_{r}}\mathrm{arccosh}\left[\frac{1}{\sqrt{{\bar{\tau }}_{r}}}\right]$$

For bond lengths higher than *λ*_lim_, setting the derivative of the debonding load (Equation (16)) to zero with respect to the crack length *a*, we find that the crack length at which the load is the highest is:

(18) $$\alpha =\lambda -\lambda_{\lim}$$

Therefore, the maximum (critical) load *F*_c_ is achieved by replacing Equation (18) into (16). Summarizing, according to EL, the maximum load is:

(19) $${\bar{F}}_{c}=\frac{F_{c}}{F_{c}^{\infty }}=\left\{ \begin{array}{l} \frac{1}{1-{\bar{\tau }}_{r}}\tanh\left[\lambda \left(1-{\bar{\tau }}_{r}\right)\right],\;\;\;\lambda \leq \lambda_{\lim}\\ \frac{1}{\sqrt{1-{\bar{\tau }}_{r}}}+{\bar{\tau }}_{r}\left(\lambda -\lambda_{\lim}\right),\;\;\;\;\;\lambda >\lambda_{\lim} \end{array}\right.$$

**Figure 4:**
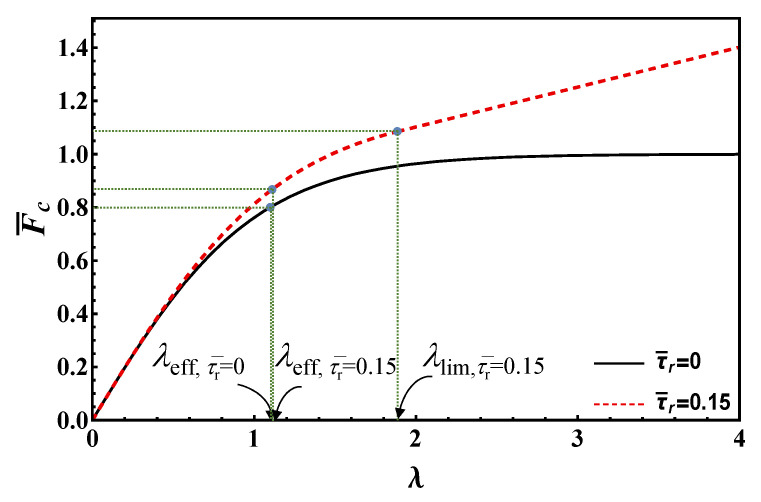
Maximum load vs. bond length according to EL for ${\bar{\tau }}_{r}=\;\;0,\;0.15$.

It is worth emphasizing that, in dimensionless form, the maximum load depends only on the normalized bond length and the ratio between residual and undamaged strengths. This feature is also shared with the following models. The maximum load vs. bond length is plotted according to Equation (19) in Figure 4 for ${\bar{\tau }}_{r}=0,\;0.15$. In this figure, the limit value of bond length, *λ*_lim_, is also illustrated for ${\bar{\tau }}_{r}=0.15$, whereas it is infinity for ${\bar{\tau }}_{r}=0$.

#### 2.1.2. Effective Bond Length

As Figure 4 clearly evidences, the maximum load strongly increases with the bond length for small values of this parameter. Then, the slope decreases, finally reaching a constant value for bond lengths higher than the limit value, see Equation (17). In contrast, in the origin the slope is proportional to the undamaged strength, *τ*_c_, for large bond lengths, the slope is proportional to the lower residual strength *τ*_r_. Therefore, one can define an effective bond length as the length above which the load increment is limited. For the EL, the *effective* bond length cannot be taken equal to the limit one (Equation (17)), since this goes to infinity when *τ*_r_ → 0. Thus, it is convenient to define the effective length as the length that tolerates *β* percent of the load at the transition point between short and long bond lengths, *λ*_lim_. Simple analytical manipulations yield:

(20) $$\lambda_{\mathrm{eff}}=\frac{1}{1-{\bar{\tau }}_{r}}\mathrm{Arctanh}\left[\beta \sqrt{1-{\bar{\tau }}_{r}}\right]$$

As shown, the effective bond length depends on *β* for EL model. As Arctanh [1] is infinity, choosing high *β* values close to unity may result in unrealistically high effective bond lengths. Thus, in order to get reasonable effective bond length estimates for any residual strength value, we opted for *β* = 80%, see Figure 4 where the effective bond lengths are highlighted for both frictionless and frictional cases (${\bar{\tau }}_{r}=0,\;0.15$).

As observed in the Introduction, despite the EL model having already been proposed in the Literature, Formula (19), providing the critical load, is original, while the effective bond length estimate (20) is supposed to be more reliable (taking friction into account) than the simpler estimate *λ*_eff_ ≅ 4 provided in [15].

### 2.2. Dugdale Model (DM)

According to DM, the interface is characterized by a constant shear stress in the cohesive process zone, equal to *τ*_c_. As the relative displacement, *s*, reaches its threshold value, *s*_f_, debonding occurs and shear stress drops to residual strength, *τ*_r_:

(21) $$\tau [s]=\left\{ \begin{array}{l} \tau_{c},\;\;\;\;\;\;\;s\leq s_{f}\\ \tau_{r},\;\;\;\;s>s_{f} \end{array}\right.$$

where:

(22) $$s_{f}=\frac{G_{c}}{\tau_{c}-\tau_{r}}$$

A view of bond-slip relation according to DM is presented in Figure 3b. Again, the shaded area represents the fracture energy.

To obtain the fracture load during the debonding, first, the minimum bond length required for a fully developed process zone should be calculated. To determine this length, traction free and no-slip at *x* = 0 are the proper boundary conditions:

(23) s[0]=0

(24) $$\sigma \left[0\right]=0\to s^{\prime }\left[0\right]=0$$

By using Equations (3) and (21) (for *s* < *s*_f_) as well as boundary conditions (23) and (24), the slip distribution is:

(25) $$s\left[x\right]=\frac{x^{2}\left(1+\rho \right)\tau_{c}\;L_{p}}{2\;E_{p}\;A_{p}}$$

**Figure 5:**
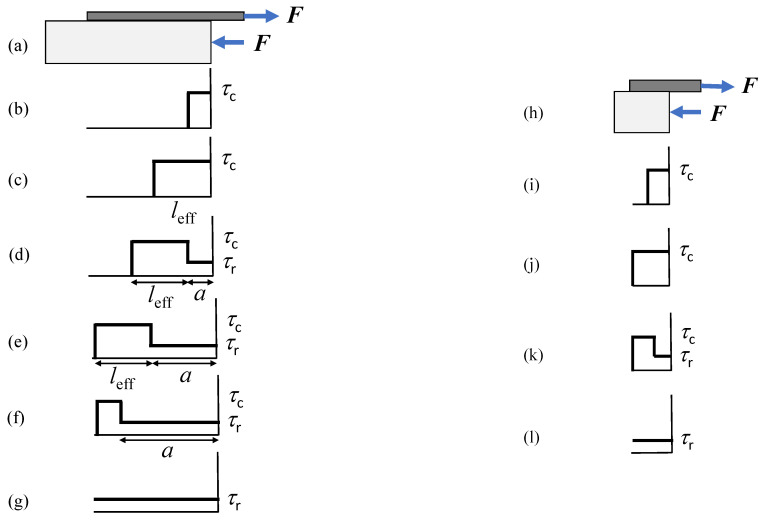
Distribution of shear stress for different stages of debonding based on DM: (**a**–**g**) for long (*l > l*_eff_), and (**h**–**l**) for short (*l< l*_eff_) bond lengths.

Now, the bond length required for a fully developed process zone can be determined using the condition that at the end of this length, the relative slip is equal to *s*_f_; see Figure 5, left side. It is worth noting that, in this figure, the area under the shear stress distribution curve represents the debonding load. On the other hand, for lengths greater than the fully developed process zone, the load increments are minor (see stages (c) to (e) in Figure 5), being attributable to the residual strength only. Consequently, this length can be considered as the effective bond length *l*_eff_ for the DM. Thus, setting *s*[*l*_eff_] = *s*_f_, by Equations (14), (22) and (25), we get:

(26) $$\lambda_{\mathrm{eff}}=\frac{l_{\mathrm{eff}}}{l_{\mathrm{ch}}}=\frac{1}{\sqrt{1-{\bar{\tau }}_{r}}}$$

Therefore, we have two different scenarios: *long* (*l > l*_eff_, left side in Figure 5) and *short* (*l < l*_eff_, right side in Figure 5) bond lengths. For long bond lengths, simple calculations show that the load during the debonding propagation (stages (c–g)) can be determined as:

(27) $$\bar{F}=\left\{ \begin{array}{l} \frac{1}{\sqrt{1-{\bar{\tau }}_{r}}}+\;\alpha \;{\bar{\tau }}_{r},\;\;\;\;\;\;\;0<\alpha \leq \lambda -\lambda_{\mathrm{eff}}\\ (\lambda -\alpha )+\alpha \;{\bar{\tau }}_{r},\;\;\;\;\;\lambda -\lambda_{\mathrm{eff}}<\alpha <\lambda \end{array}\right.\;\;$$

#### Maximum Load vs. Bond Length

From Equation (27), it is clear that the maximum load is achieved when *α* equals (*λ − λ*_eff_), i.e., at stage (e) in Figure 5 (left column). On the other hand, the maximum is reached at debonding onset for short bond lengths, i.e., at stage (j), right column in Figure 5. Hence, we have:

(28) $${\bar{F}}_{c}=\frac{F_{c}}{F_{c}^{\infty }}=\left\{ \begin{array}{l} \lambda ,\;\;\;\;\;\;\;\;\;\;\;\;\lambda \leq \lambda_{\mathrm{eff}}\\ \frac{1}{\sqrt{1-{\bar{\tau }}_{r}}}+{\bar{\tau }}_{r}\left(\lambda -\lambda_{\mathrm{eff}}\right),\;\;\;\lambda >\lambda_{\mathrm{eff}} \end{array}\right.$$

**Figure 6:**
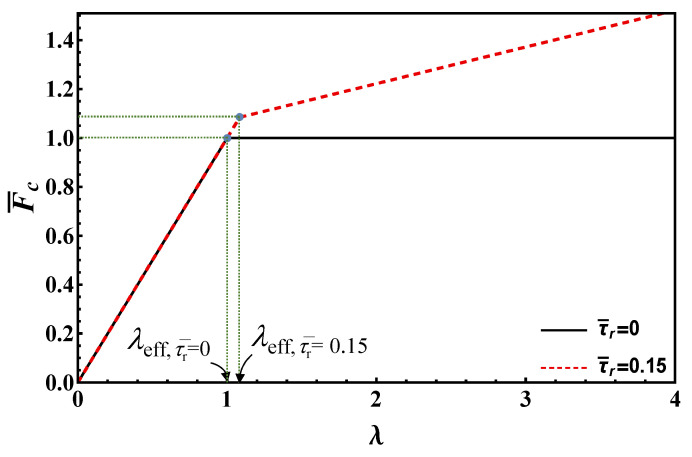
Maximum load vs. bond length according to DM for ${\bar{\tau }}_{r}=\;\;0,\;0.15$.

In Figure 6, the maximum load vs. bond length for ${\bar{\tau }}_{r}=0,\;0.15$ is shown based on Equation (28), whereas the effective bond length *λ*_eff_ is also illustrated. Note that the maximum load corresponding to the limit/effective bond length, according to EL and DM, is the same and higher than $F_{c}^{\infty }$ (if friction is present), respectively.

### 2.3. Rigid-Linear Softening Model (RL)

According to RL (Figure 3c), the interface is characterized by a (linear) softening from *τ*_c_ to *τ*_r_ as the relative displacement *s* increases from 0 to *s*_f_. The corresponding bond-slip law for this model is:

(29) $$\tau [s]=\left\{ \begin{array}{l} \tau_{c}-(\tau_{c}-\tau_{r})\frac{s}{s_{f}},\;\;\;s\leq s_{f}\\ \tau_{r},\;\;\;\;\;\;\;\;s>s_{f} \end{array}\right.$$

For the RL model, the final relative displacement writes:

(30) $$s_{f}=\frac{2\;G_{c}}{\tau_{c}-\tau_{r}}$$

As for DM, to compute the load during debonding, the minimum length for a fully developed softening zone, *l*_eff_, has to be determined. The boundary conditions of the problem are no-slip and traction-free at *x* = 0, see Equations (23) and (24). By inserting the RL constitutive law (29). *s* < *s*_f_) into the governing equation, Equation (3), the relative slip and shear stress along the interface are:

(31) $$s[x]=s_{f}\frac{1-\cos\left[\frac{x}{l_{\mathrm{ch}}}(1-{\bar{\tau }}_{r})\right]}{1-{\bar{\tau }}_{r}}$$

(32) $$\tau [x]=\tau_{c}\cos\left[\frac{x}{l_{\mathrm{ch}}}\left(1-{\bar{\tau }}_{r}\right)\right]$$

**Figure 7:**
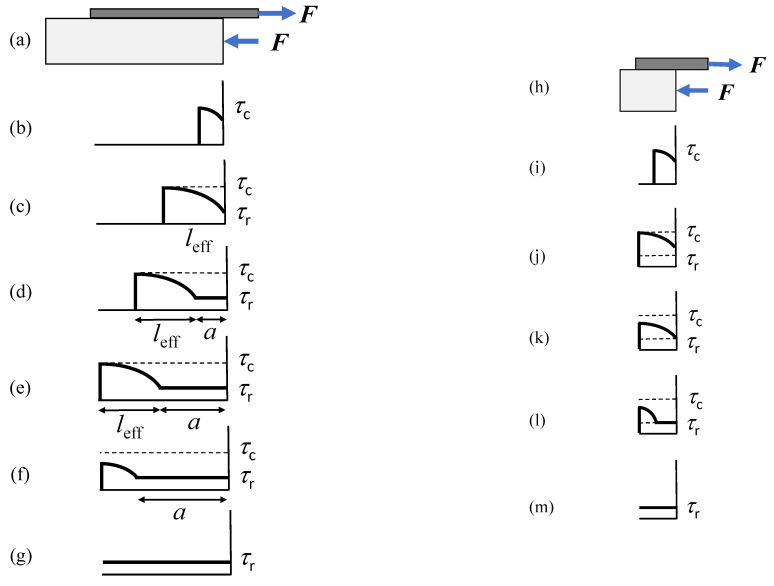
Distribution of the shear stresses for different stages of debonding based on the RL model: (**a**–**g**) for long, and (**h**–**m**) for short bond lengths.

In Figure 7, the shear stresses for different stages of debonding based on the RL model are plotted.

Also, for the RL model, the bond length required for a fully developed softening zone, *l*_eff_, can be calculated setting *s*[*l*_eff_] = *s*_f_ in Equation (31) (or, equivalently, *τ*[*l*_eff_] = *τ*_r_ in Equation (32)). Thus:

(33) $$\lambda_{\mathrm{eff}}=\frac{l_{\mathrm{eff}}}{l_{\mathrm{ch}}}=\frac{\arccos\left[{\bar{\tau }}_{r}\right]}{1-{\bar{\tau }}_{r}}$$

For long bond lengths (*l > l*_eff_, left side in Figure 7), during debonding (stages (c–e)), the applied load can be computed as:

(34) $$\bar{F}=\frac{F}{F_{c}^{\infty }}=\;\frac{L_{p}\int_{0}^{l_{\mathrm{eff}}}\tau [x]\;dx+\tau_{r}\;a\;L_{p}}{\tau_{c}\;l_{\mathrm{ch}}\;L_{p}}=\sqrt{\frac{1+{\bar{\tau }}_{r}}{1-{\bar{\tau }}_{r}}}+\;\alpha \;{\bar{\tau }}_{r},\;\;0<\alpha \leq \lambda -\lambda_{\mathrm{eff}}$$

For stages (e) to (g), i.e., *α* > *λ*−*λ*_eff_, it is necessary first to solve Equation (3) in the softening zone with the first row in Equation (29). Boundary conditions are represented by Equation (24) and:

(35) $$s\left[l-a\right]=s_{f}$$

In this way we get *s*[*x*] for 0 < *x* < *l*−*a*. Its derivative at the right edge is:

(36) $$s^{\prime }\left[l-a\right]=\frac{s_{f}}{l_{\mathrm{ch}}}{\bar{\tau }}_{r}\;\tan\left[\frac{l-a}{l_{\mathrm{ch}}}\left(1-{\bar{\tau }}_{r}\right)\right]$$

Then, we solve Equation (3) in the frictional zone, i.e., with the second row in Equation (29) and boundary conditions (35) and (36), since the stress and the displacement must be continuous at *x* = *l*−*a*. In this way, we get *s*[*x*] for *l*−*a* < *x* < *l*. Evaluating its derivative at the loaded end, using Equation (4), we finally get the load for stages (e–g):

(37) $$\bar{F}=\frac{{\bar{\tau }}_{r}}{1-{\bar{\tau }}_{r}}\tan\left[\left(\lambda -\alpha \right)\left(1-{\bar{\tau }}_{r}\right)\right]+\alpha \;{\bar{\tau }}_{r}\;,\;\;\lambda -\lambda_{\mathrm{eff}}<\alpha <\lambda$$

Equations (34) and (37) define the load during debonding according to RL. As such, they are analogous to Equation (27) for DM, and Equation (16) for EL.

#### Maximum Load vs. Bond Length

For short bond lengths, it is easily seen from Figure 7 (right column) that the maximum is achieved at stage (j), i.e., when the whole interface softens and *τ*[0] = *τ*_c_. By Equation (32):

(38) $$F_{c}=\;L_{p}\int_{0}^{l}\tau [x]\;dx=\frac{\sin\left[\lambda \left(1-{\bar{\tau }}_{r}\right)\right]}{1-{\bar{\tau }}_{r}}\tau_{c}\;l_{\mathrm{ch}}\;L_{p}$$

On the other hand, for long bond lengths, *λ* > *λ*_eff_, the maximum load occurs at stage (e), and its value is achieved by replacing *α* = *λ* − *λ*_eff_ into either Equation (34) or Equation (37). Summarizing, we have:

(39) $${\bar{F}}_{c}=\frac{F_{c}}{F_{c}^{\infty }}=\left\{ \begin{array}{l} \frac{1}{1-{\bar{\tau }}_{r}}\sin\left[\lambda \left(1-{\bar{\tau }}_{r}\right)\right],\;\;\;\;\;\;\lambda \leq \lambda_{\mathrm{eff}}\\ \sqrt{\frac{1+{\bar{\tau }}_{r}}{1-{\bar{\tau }}_{r}}}+{\bar{\tau }}_{r}\;(\lambda -\lambda_{\mathrm{eff}}),\;\;\;\;\lambda >\lambda_{\mathrm{eff}} \end{array}\right.$$

**Figure 8:**
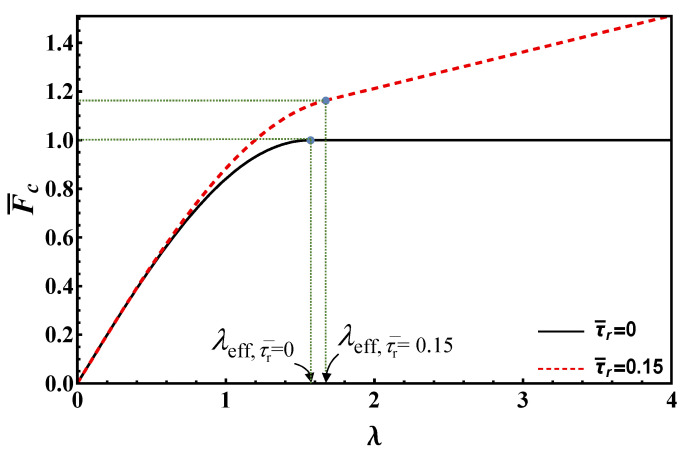
Maximum load vs. bond length according to RL for ${\bar{\tau }}_{r}=\;\;0,\;0.15$.

Equation (39) is plotted in Figure 8 to illustrate the behavior of maximum load vs. bond length according to RL for ${\bar{\tau }}_{r}=0,\;0.15$. Note that the maximum load corresponding to the effective bond length (first term in the second row in Equation (39)) according to RL is higher than the ones provided by EL and DM.

### 2.4. Rigid-Finite Fracture Mechanics Model (RF)

In this section, a fracture mechanics based model called FFM is employed to tackle the debonding process in the direct shear test. According to FFM [17], the crack is assumed to grow by a discrete amount Δ. Two conditions have to be fulfilled for crack growth. The first is the discrete energy balance:

(40) $$L_{p}\;\int_{a}^{a+\Delta }G(a^{\prime })\;da^{\prime }\;\geq G_{c}\;L_{p}\;\Delta$$

For the sake of simplicity and of comparison with the rigid cohesive zone models (i.e., DM and RL), for this model, we consider a rigid interface too. Thus, from Equations (9) and (11) and letting *k* → ∞, we get:

(41) $$G=\frac{{\left(\tau_{\max}-\tau_{r}\right)}^{2}}{2\;k}\;\;\underset{k\to \infty }{=}\;\;\frac{1+\rho }{2E_{p}\;A_{p}\;L_{p}}\;{\left(F-a\;L_{p}\;\tau_{r}\right)}^{2}$$

Substituting Equation (41) into (40), we get, in dimensionless form:

(42) $${\bar{F}}^{2}-{\bar{\tau }}_{r}(2\;\alpha +\delta )\;\bar{F}+{\bar{\tau }}_{r}^{2}(3\;\alpha^{2}+3\;\alpha \;\delta +\delta^{2})\geq 1$$

where *δ* = Δ/*l*_ch_. The second condition to be fulfilled is a stress requirement: the average stress acting on the crack advance Δ must be higher than the interfacial strength *τ*_c_. Since the interface is rigid, the force in the plate is transferred to the block in a pointwise manner, i.e., abruptly at the crack tip (*x* = *l* − *a*). Hence, the stress condition reads:

(43) $$\frac{F-\tau_{r}\;a\;L_{p}}{L_{p}\;\Delta }\geq \tau_{p}$$

By some analytical manipulations of Equation (42), and casting in dimensionless form Equation (43), the debonding load can be determined as the minimum load satisfying the following system of inequalities:

(44) $$\left\{ \begin{array}{l} \bar{F}\geq \left(\left(\alpha +\delta /2\right){\bar{\tau }}_{r}+\sqrt{1-{\left({\bar{\tau }}_{r}\delta \right)}^{2}/12}\right)\;H\left[\left(\lambda -\alpha \right)-\delta \right]\\ \bar{F}\geq \delta +{\bar{\tau }}_{r}\;\alpha \end{array}\right.$$

where the Heaviside function, H[⋅], has been introduced because the energy condition is always fulfilled in case of complete failure, i.e., Δ = *l* − *a*. 

**Figure 9:**
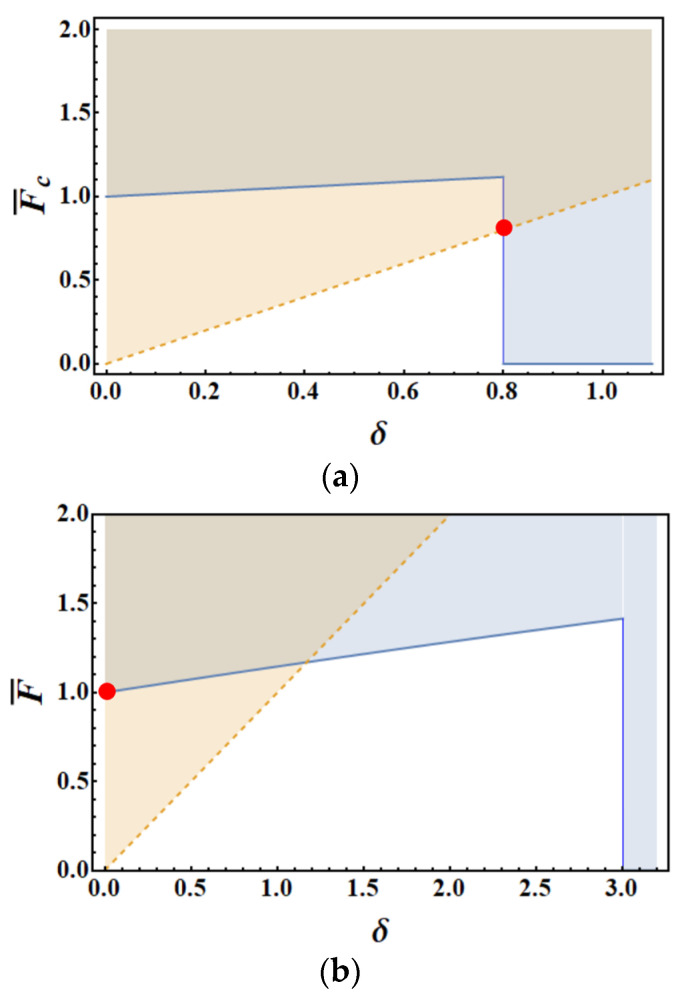

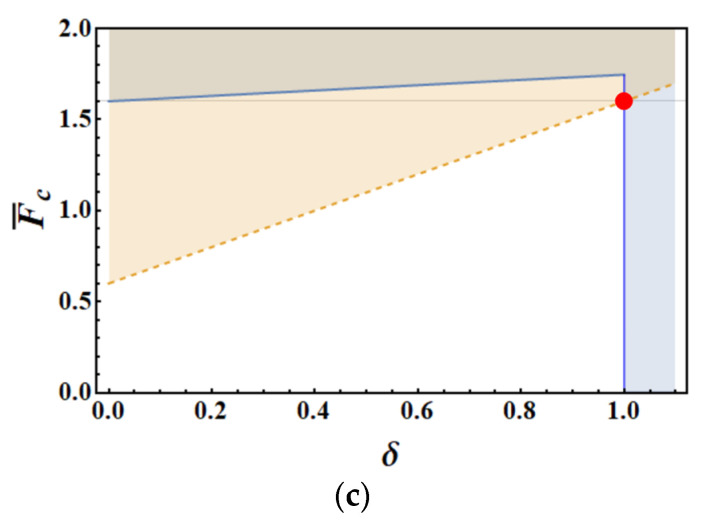
Graphical representation of the inequality system (44): continuous lines show energy condition, first inequality; dashed lines illustrate stress requirement, second inequality. The dot highlights the minimum load, thus identifying the debonding load and the corresponding crack growth. Normalized load vs. crack advance according to RF model for (**a**) a short bond length (*λ* = 0.8) at debonding onset (*α* = 0); (**b**) a long bond length (*λ* = 3) at debonding onset (*α* = 0, stable crack growth); (**c**) a long bond length (*λ* = 3) when the load is maximum (*α* = 2) and the crack growth turns unstable. We assumed ${\bar{\tau }}_{r}=0.3$.

Looking for the maximum load $F_{c}^{}$ during the debonding process, it is easy to check that two cases can be met. In the first one, occurring for short bond lengths, *λ* < 1, the maximum load is achieved at debonding onset, *α* = 0, see Figure 9a. Accordingly,${\bar{F}}_{c}$= *δ* = *λ* and crack propagation is obviously unstable (under load control), being an abrupt debonding crack predicted all over the interface. In the latter case, occurring for long bond lengths (*λ* > 1), the minimum of the system in Equation (44) is achieved for an infinitesimal crack growth (*δ* = 0) and for a dimensionless load equal to unity, see Figure 9b. However, in such a case, the debonding process is stable, since the debonding load increases along with the crack length *α*. Figure 9c shows, however, that crack growth becomes unstable when the debonding crack *α* reaches the value (*λ* − 1) and the crack increment for which the load is minimum jumps from *δ* = 0 to *δ* = 1, i.e., the crack advance coincides with the ligament. The corresponding load reveals itself to be the maximum one, and is equal to ${\bar{F}}_{c}$= 1 +${\bar{\tau }}_{r}$(*λ*−1), see Figure 9c.

From the above observations, it is clear that for long bond lengths, the load during debonding is given by the energy condition (first equation in system (44)) with infinitesimal crack increment (*δ* = 0) as long as *α* < *λ* − 1, and by the stress condition (second equation in system (44)) with crack advance equal to the ligament (*δ* = *λ* − *α*) for *α* > *λ* − 1:

(45) $$\bar{F}=\left\{ \begin{array}{l} 1+\alpha \;{\bar{\tau }}_{r},\;\;\;\;\;\alpha \leq \lambda -1\\ (\lambda -\alpha )+\alpha \;{\bar{\tau }}_{r},\;\;\;\;\alpha >\lambda -1 \end{array}\right.$$

Note that the second row in Equation (45) coincides with the one valid for DM, Equation (27).

#### Maximum Load vs. Bond Length

From the previous analysis, the maximum load vs. bond lengths reads:

(46) $${\bar{F}}_{c}=\frac{F_{c}}{F_{c}^{\infty }}=\left\{ \begin{array}{l} \lambda \;,\;\;\;\;\;\;\lambda \leq 1\\ 1+{\bar{\tau }}_{r}\;(\lambda -1),\;\lambda >1 \end{array}\right.$$

**Figure 10:**
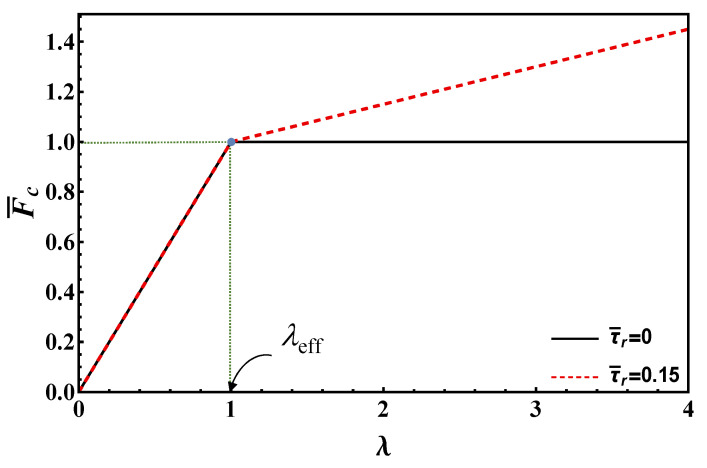
Maximum load vs. bond length according to RF for ${\bar{\tau }}_{r}=\;\;0,\;0.15$.

Equation (46) is plotted in Figure 10 to illustrate the maximum load vs. bond length according to RF for ${\bar{\tau }}_{r}=0,\;0.15$. It is clear that, according to RF model, the effective bond length is always equal to unity (*λ*_eff_ = 1), and thus is independent of the residual strength. The maximum load corresponding to the effective bond length is equal to $F_{c}^{\infty }$, and thus is lower than those provided by the previous models.

## 3. Results and Discussions

In this section, some parametric studies are performed on the effect of residual strength and bond length on the maximum debonding load. The effective bond length relations are recalled and investigated in more detail, and the evolution of the load during the debonding process is assessed according to the different models. Finally, theoretical predictions are examined and compared with the experimental data available in the literature.

### 3.1. Maximum Debonding Load vs. Bond Length

**Figure 11:**
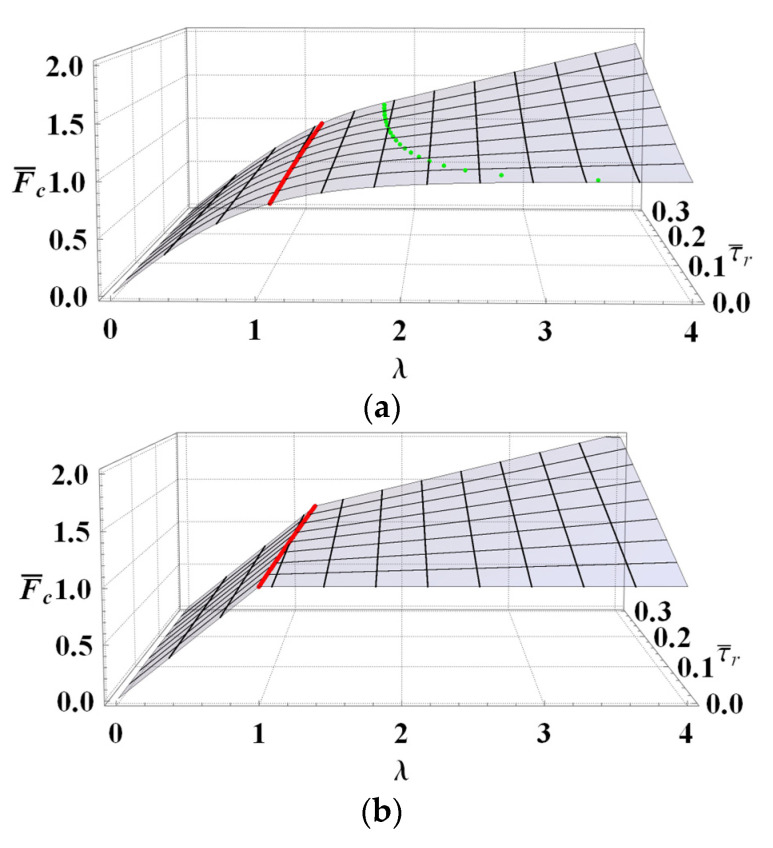

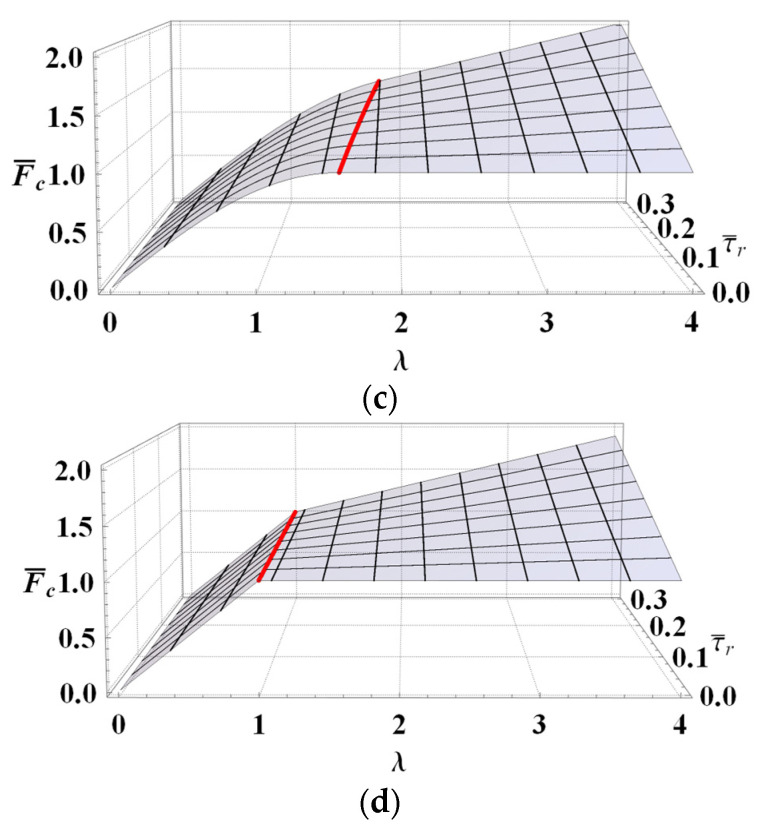
Maximum debonding load vs. bond length and residual strength according to: (**a**) EL, (**b**) DM, (**c**) RL, (**d**) RF. Effective bond lengths are evidenced with solid lines. Note that for the EL model *β* = 0.8 and the limit bond length for this model is also highlighted with dots in (**a**).

The maximum debonding load is depicted in Figure 11a–d as a function of residual strength and bond length for EL, DM, RL and RF, respectively. The effective bond length for each model is illustrated with solid lines, while the dots in Figure 11a show the evolution of *λ*_lim_ for EL.

For the case of ${\bar{\tau }}_{r}=0$ and long bond lengths, DM, RL and RF predict ${\bar{F}}_{c}$= 1, while for EL, an asymptotic behavior towards unity can be seen. Equivalently, for the EL model, the bond length value *λ*_lim_, separating short and long bond joint solutions, goes to infinity as the residual strength vanishes. As such, the limit value cannot be used as an effective bond length estimate for the EL model, while it can for DM, RL and RF.

All in all, Figure 11 clearly shows that each graph can be divided into two different parts via *λ*_eff_. In the left part, for increasing bond lengths, the load increment is strong, whereas it is weak in the right part. It is worthwhile to note that the left parts are determined via the first row of Equations (19), (28), (39) and (46), while the right parts are plotted using the relationships in the corresponding second row. Also, the effect of residual strength on maximum debonding load is higher for long bond lengths than for short ones.

**Figure 12:**
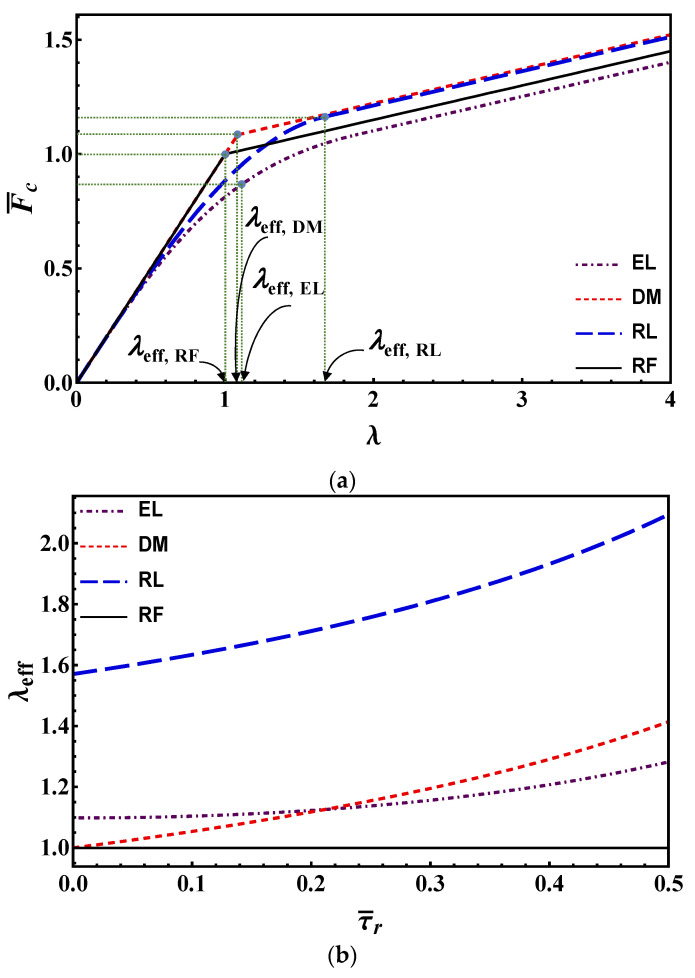
(**a**) Maximum debonding load vs. bond length according to EL, DM, RM and RF models for ${\bar{\tau }}_{r}=\;\;0.15$; (**b**) effective bond length vs. residual strength. For EL, *β* = 0.8.

For a better understanding and in order to draw a comparison, the estimations of maximum debonding load according to different models are plotted in Figure 12a for a constant residual strength (${\bar{\tau }}_{r}=0.15$). As shown, all the models for very short bond lengths predict equivalent maximum debonding load. On the other hand, for long bond lengths, predictions by DM and RL are almost the same, RF is in the middle while EL presents lower estimations for the maximum debonding load. Note also that the slope of the tangent in the origin is proportional to the undamaged shear strength *τ*_c_ (1 in the dimensionless plot), whereas the slope of the linear branch in the frictional stage is proportional to *τ*_r_ (${\bar{\tau }}_{r}$ in the dimensionless plot).

Finally, in case no information is available for the shape of the bond-slip law, it may be advised to employ the EL model, as it yields the most conservative estimates for the maximum load. The other models might overestimate it.

### 3.2. Effective Bond Length

For the sake of clarity, it is good to recall expressions for the effective bond length in their dimensional forms, according to the EL, DM, RL and RF models, respectively:

(47) $$l_{\mathrm{eff},\;\mathrm{EL}}=\frac{\mathrm{arctanh}\left[\beta \sqrt{1-\tau_{r}/\tau_{c}}\right]}{1-\tau_{r}/\tau_{c}}\;\;\frac{1}{\tau_{c}}\sqrt{\frac{2\;G_{c}\;E_{p}\;A_{p}}{(1+\rho )L_{p}}}$$

(48) $$l_{\mathrm{eff},\;\mathrm{DM}}=\frac{1}{\sqrt{1-\tau_{r}/\tau_{c}}}\;\;\frac{1}{\tau_{c}}\sqrt{\frac{2\;G_{c}\;E_{p}\;A_{p}}{(1+\rho )L_{p}}}$$

(49) $$l_{\mathrm{eff},\;\mathrm{RL}}=\frac{\arccos\left[\tau_{r}/\tau_{c}\right]\;}{1-\tau_{r}/\tau_{c}}\;\;\frac{1}{\tau_{c}}\sqrt{\frac{2\;G_{c}\;E_{p}\;A_{p}}{(1+\rho )L_{p}}}$$

(50) $$l_{\mathrm{eff},\;\mathrm{RF}}=l_{\mathrm{ch}}=\;\;\frac{1}{\tau_{c}}\sqrt{\frac{2\;G_{c}\;E_{p}\;A_{p}}{(1+\rho )L_{p}}}$$

Note that the dimensional part is the same for all equations, which therefore differ only because of the dimensionless factor, depending on the residual to undamaged shear strength ratio.

For the EL model, the value of *β* was set to 0.8 in Figure 12b. Beyond the advantages already highlighted in Section 2.1.2, this value ensures a monotonically increasing effective bond length, i.e., a physically consistent trend and in agreement with the other models. It is worth noting that Equation (49) can be seen as a straightforward generalization of the formula proposed by the Italian Standards CNR-DT 200 R1/2013 [44] in order to take the effect of the residual strength (and the mechanical fraction of reinforcement) into account. In fact, by setting *τ*_r_ and *ρ* to zero (and introducing the plate thickness *t*_p_ = *A*_p_/*L*_p_) in Equation (49), the CNR-DT 200 R1/2013 effective bond length expression is recovered.

(51) $$l_{\mathrm{eff},\mathrm{CNR}-\mathrm{DT}}=\frac{\pi }{\tau_{c}}\sqrt{\frac{G_{c}\;E_{p}\;t_{p}}{2}}$$

In Figure 12a, the effective bond length estimates, Equations (47)–(50), according to the four models are highlighted in the maximum load vs. bond length plot (for a given residual strength value), whereas in Figure 12b they are plotted as a function of the normalized residual strength. Figure 12b shows that for allmodels, except RF, the effective bond length increases with increasing residual strengths, while it is constant for RF. The RL is the most conservative among the proposed criteria, providing the highest estimate for the effective bond length.

Finally, with regard to neglect the residual strength (which might be a considerable value, especially for FRCMs), one underestimates both the debonding load and the effective bond length. Thus, using expressions like Equation (13) or Equation (51), the strengthening system is not used to its full potential.

### 3.3. Debonding Load vs. Relative Crack Length

**Figure 13:**
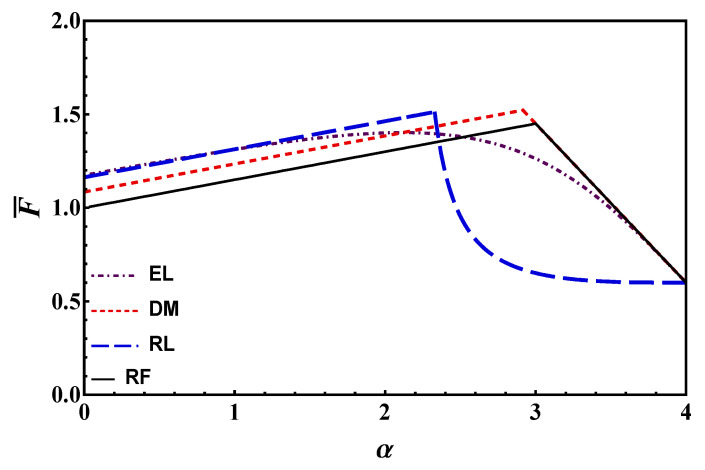
Debonding load as a function of debonding crack length *α* for *λ* = 4 and ${\bar{\tau }}_{r}=\;\;0.15$.

For the sake of simplicity, in this paper, we derived the expression of the load during debonding only for long bond lengths. The corresponding plot is provided in Figure 13 according to the different models, i.e., by Equations (16), (27), (34), (37) and (45). It is seen that all models except RF estimate a normalized load higher than unity at *α* = 0, i.e., the effect of residual strength is present also at the onset of debonding. DM and RF show the same behavior for debonding process: a linear increase, and then a linear decrease. Note that predictions by RF are lower in the stable debonding branch. Also, RL shows a linear increase of the load in the stable branch, followed by a dramatic decrease after the maximum load has been reached. The dramatic decrease of force when *α* > *λ−λ_eff_* can be explained by looking at Figure 7. Because of the (linear) softening, at the beginning of the softening-frictional stage (stages (e) and (f) in Figure 7) the reduction in load is higher than that observed through other models (e.g., stages (e) and (f) in Figure 5). EL provides the lowest maximum load, and a smooth load variation during debonding.

Figure 13 also confirms the trend in Figure 12a. Therefore, ordering the models from the largest to the smallest maximum load estimate provided, we have DM, RL, RF, EL. This trend is illustrated in Figure 12a (for a fixed—long—bond length).

### 3.4. Comparison with Experimental Data

In order to validate the presented models, experimental data of direct single lap shear test for polyparaphenylene benzobisoxazole (PBO) FRCM-to-concrete joint were taken from the literature [13], see Figure 1d as reference. For these experiments, concrete blocks with 125 mm × 125 mm cross-section were used. Each fiber of the net had a cross-section of about 5 mm × 0.092 mm with an elastic modulus of 206 GPa, settled between two 4 mm thick cementitious matrix layers (as the elastic modulus of the matrix is not mentioned in the reference paper, we assumed it to be equal to 30 GPa [45]).

The experimental results of three different bond widths, i.e., 43, 60, 80 mm, and five different bond lengths, i.e., *l* = 100, 150, 200, 250, 330, 450 mm, were employed to assess the accuracy of the models in estimating the maximum debonding load. Failure mainly occurred by debonding between the PBO net and the cementitious matrix. Thus, we considered *A*_p_ ~ *n* × 5 mm × 0.092 mm and *L*_p_ ~ *n* × 2 × 5 mm, where *n* is the number of PBO longitudinal fibers, i.e., 5, 7, and 9 for a reinforcement-to-substrate width equal to 43, 60 and 80 mm, respectively.

The central PBO fiber of four specimens (all with a bond length equal to 330 mm) was equipped with strain gauges. By these measurements, in a subsequent paper, D’Antino et al. [12,13] were able to determine an average bond-slip law whose parameters were G_c_ = 0.481 N/mm, *τ*_c_ = 0.77 MPa and *τ*_r_ = 0.06 MPa. Note that the authors used a different definition of fracture energy (namely, the total area under the tau-slip curve up to *s*_f_), so that their fracture energy corresponded to a value equal to 0.387 N/mm in the present model. Hence, for the above experimental data, from Equations (13)–(15) we get ${\bar{\tau }}_{r}=0.078$, $F_{c}^{\infty }/n=819.3\;N$ and *l*_ch_ = 106.4 mm.

**Figure 14:**
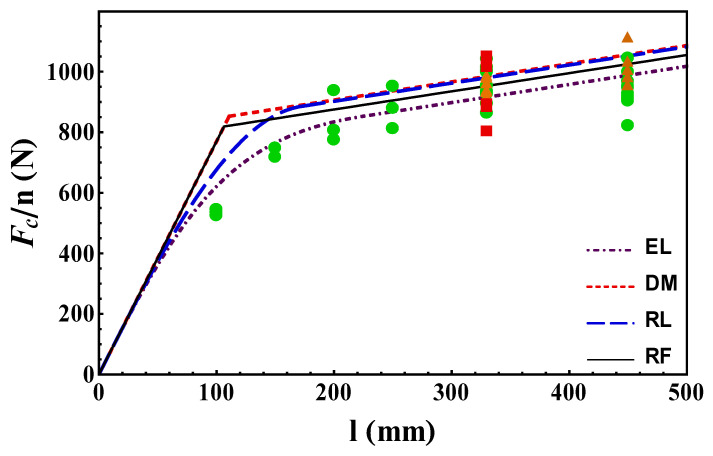
Maximum debonding load for different bond lengths and widths. Dot-dashed line, short dashed line, long dashed line and solid line refer to estimations using EL, DM, RL and RF, respectively. The experimental data from [13] are shown by markers, whose shape refers to different widths, i.e., squares, circles, and triangles are representative for widths of 43, 60, 80 mm.

A comparison between the experimental data and the analytical predictions in terms of failure load (per unit fiber) vs. bond length is shown in Figure 14. The theoretical results are illustrated by lines, whereas the experimental results referring to widths of 43, 60, 80 mm are plotted by squares, circles and triangles, respectively.

It is remarkable to see that all models were able to catch the experimental data trend rather satisfactorily. The agreement between theory and experiments is even more valuable if we observe that the interface parameters were not fitted for the matching, but derived from strain and displacement measures performed on just one geometry (more precisely that with bond length 330 mm). In other words, by testing one bond length, the models were able to predict the failure for any bond length (and thickness). This proves the soundness of the presented approaches (beyond the high accuracy of the experimental measures provided in [12]).

In greater detail, for the bond lengths close to the transition length *l*_ch_, DM and RF predictions were rather poor, since the experimental data showed a smooth transition between short and long bond lengths, whereas the EL and RL models seemed to catch this transition. EL predictions for short bond lengths were the best, while RF was the model that best matched experiments for long bond lengths. This observation, along with its simplicity, proves the effectiveness of the FFM approach regarding the problem at hand. Finally, regarding the data related to different reinforcement widths, Figure 14 shows that although the models were one-dimensional, they take the width effect into account reasonably well.

To compare the results of each model for different bond lengths in more detail, the corresponding percentage error is presented in Table 1.

Table 2 also presents the correlation coefficient, mean squared error, and effective bond length for each model to better evaluate the performance of each model.

It should be noted that the results of effective bond length are in agreement with those calculated in [46] using the finite difference method.

Finally, it is worth noting that if failure loads are recorded only in an experimental campaign, the interface parameters can be determined by a best fitting procedure provided that data are available for several (short and long) bond lengths. Without entering into the details of a best fitting procedure, we just want to observe that, starting from the maximum load vs. bond length experimental curve, *τ*_c_ can be recovered from its slope in the origin, *τ*_r_ from its slope for long bond lengths, and G_c_ from the position of the knee between short and long bond length asymptotes.

## 4. Conclusions

By means of four (three Cohesive and one FFM) one-dimensional, three-parameter interface models (*τ*_c_, *τ*_r_, and G_c_), the effect of residual strength on the mechanics of debonding in the direct shear tests was investigated, both for short and long bond lengths. The laws utilized here refer to different interface behaviors, i.e., EL models the interface as a bed of linear elastic-purely brittle springs, while DM considers a constant shear stress distribution along the process zone, RL assumes a softening along the process zone and RF is based on finite fracture mechanics. Thanks to the relative simplicity of these approaches, relevant quantities were determined in closed-form expressions, which are handy for design purposes of strengthening of beams. It is noteworthy that the effective bond length formula provided by the rigid-linear softening model is a straightforward generalization of cases where the residual strength is non-negligible regarding the one proposed by Italian design guidelines.

Then, the proposed models were compared and validated against available data in the literature for FRCM-to-concrete joints, while all of the models could predict failure loads for different bond widths and lengths with reasonable accuracy.

It is shown that three models yielded higher effective bond length values when residual strength was considered (no increment according to RF). Thus, design rules should be more demanding in terms of minimum bond length. On the other hand, engineers can depend upon a higher load value in the reinforcement. This means that friction and interlocking between the fiber/reinforcement and the matrix/substrate have an overall positive effect on the strengthening system.

The main disadvantage of all models, except for EL, is that they exhibit a sharp transition from short to long bond lengths, a transition that appears smooth in the experimental data. It is expected that this problem will be solved by taking the interface elastic stiffness into account, thus retaining the best of elastic-purely brittle and rigid-softening or rigid-finite fracture mechanics approaches.

Future developments will include stiffness as a fourth independent interface parameter, being either infinite or dependent in the approaches proposed herein. Such a goal has been partly already achieved in the domain of cohesive modelling [8,9], but still has to be developed for the FFM approach.

## Figures and Tables

**Table 1:** Percentage error between analytical and (average) experimental maximum loads [13] for each bond length and each model. Error = 100(model-experiment)/experiment.

	Bond Length (mm)	100	150	200	250	330	450
EL	Percentage error	14	3	−1	−2	−5	1
DM		40	19	7	6	2	9
RL		23	15	7	5	1	8
RF		40	15	4	2	−1	5

**Table 2:** The correlation coefficient, mean squared error, and effective bond length for each model.

Model	EL	DM	RL	RF
Correlation coefficient	0.827	0.797	0.827	0.776
Mean squared error (N)	72	98	85	143
Effective bond length (mm)	120	113	176	109

## Data Availability

Not applicable.
